# Effect of Coffee Grounds/Coffee Ground Biochar on Cement Hydration and Adsorption Properties

**DOI:** 10.3390/ma17040907

**Published:** 2024-02-15

**Authors:** Yang Chen, Rongxin Guo, Feiyue Ma, Haoxue Zhou, Miao Zhang, Qianmin Ma

**Affiliations:** 1 Faculty of Civil Engineering and Mechanics, Kunming University of Science and Technology, Kunming 650500, China; 18206718572@163.com (Y.C.); guorx@kmust.edu.cn (R.G.); mfy@stu.kust.edu.cn (F.M.); zhouhaoxue198@163.com (H.Z.); miaozhang1007@163.com (M.Z.); 2 Yunnan Key Laboratory of Disaster Reduction in Civil Engineering, Kunming 650500, China; 3 International Joint Laboratory for Green Construction and Intelligent Maintenance of Yunnan Province, Kunming 650500, China

**Keywords:** chloride ion adsorption ability, formaldehyde adsorption ability, compressive strength, coffee ground biochar, cement

## Abstract

Taking advantage of the strong adsorption characteristics of coffee grounds (CGs) and coffee ground biochar (CGB), this research employed equal amounts of 2%, 4%, 6%, and 8% CGs and CGB to replace cement. This study thereby examined the impacts of CGs and CGB on cement compressive strength, as well as their abilities to adsorb chloride ions and formaldehyde. X-ray powder diffraction (XRD), Fourier transform infrared spectroscopy (FTIR), thermogravimetric analysis (TG−DTG), scanning electron microscopy (SEM), and X−ray photoelectron spectroscopy (XPS) were employed to investigate the hydration mechanism and characterize the microscopic structure. The results show the following: (1) The presence of a substantial quantity of organic compounds in CGs is found to have an adverse effect on both the compressive strength and hydration degree of the sample. The use of CGB after high-temperature pyrolysis of phosphoric acid can effectively improve the negative impact of organic compounds on the sample. (2) The addition of CGs reduces the adsorption of chloride ions by cement, primarily due to the presence of fewer hydration products. However, when CGB was incorporated into cement, it enhanced the ability to adsorb chloride ions. (3) Cement containing 8% CGB content can slightly enhance the adsorption of formaldehyde. However, the cement sample with 8% CGB content exhibited the most significant ability to adsorb formaldehyde.

## 1. Introduction

As the global economy and population continue to expand, the generation of solid waste is on the rise. As global annual coffee production exceeds 10.5 million tons [1], coffee grounds (CGs) have emerged as a prevalent type of solid waste. Due to the elevated organic content in CGs, there is a significant risk of producing gases, such as methane and carbon dioxide, during incineration [2]. Therefore, it is necessary to find an effective way to treat/reuse CGs.

It has been reported that because the surfaces of CGs have an irregular porous structure and CGs contain a variety of functional groups, such as hydroxyl groups, carboxyl groups, etc., CGs show better adsorption advantages [3]. The coffee ground biochar (CGB) obtained through the acidification and high-temperature treatment of CGs not only introduces phosphorus-containing groups but also enhances their porosity and specific surface area. This further augments CGB’s adsorption capacity [4]. CGs and CGB exhibit remarkable adsorption properties. Combining CGs/CGB with cementitious materials has the potential to enhance the adsorption capacity of cement, including in terms of chloride ion and formaldehyde adsorption. Additionally, this integration can contribute to the improved durability of cement concrete in marine environments and enhance indoor environmental quality in buildings. Furthermore, it opens avenues for expanding the reuse of CGs, thereby further elevating the recycling rate of this waste material.

In cementitious materials, chloride ions combine through two distinct mechanisms. One involves chemical combination via hydration products, leading to the formation of compounds such as Friedel’s (FS) salt or Kuzel’s salt. The other mechanism involves hydration. The product is physically combined through the adsorption process via C-S-H gel [5,6]. Prior research has indicated that the primary mechanism through which cement-based materials eliminate formaldehyde is the adsorption of formaldehyde molecules by the inherent pore structure of the cement itself. Simultaneously, the cured formaldehyde molecules undergo oxidation and decomposition into H_2_O and CO_2_ in the presence of strong hydroxyl radicals. Hadj et al. [7] attempted to enhance the formaldehyde adsorption of cement by incorporating TiO_2_. Their findings revealed that when the TiO_2_ content was 5%, the photocatalytic degradation rate of formaldehyde by cement reached its optimal level. Verriele et al. [8] conducted a characterization of the formaldehyde adsorption capacity of lime–cement–gypsum composite materials. Their study revealed that the composite material can convert formaldehyde into gaseous methanol and adsorbable formate, resulting in a substantial reduction in indoor formaldehyde concentrations. On the other hand, Boonamnuayvitaya et al. [9] used a ZnCl reagent and nitrogen impregnation to activate CGs to prepare CGB and found that the hydrophilic functional group of the CGB has a high adsorption capacity for formaldehyde. Ahn et al. [10] discovered that the pyrolysis of sludge and CGs combined resulted in the formation of a novel type of metal-embedded CGB. This CGB exhibited an impressive formaldehyde adsorption capacity of 1811 mg/g, showcasing its effectiveness in the removal of formaldehyde.

In summary, both CGs and CGB demonstrate remarkable adsorption capabilities. Integrating them with cementitious materials holds the potential to enhance the adsorption capacity of these materials, including with respect to aspects such as chloride ion adsorption and formaldehyde adsorption. However, there is a scarcity of relevant studies on this subject. Therefore, in this study, we aimed to investigate the adsorption capacity and mechanism of composite materials comprising cement and both CGs and CGB for chloride ions and formaldehyde. The findings can provide scientific theoretical guidance for the adsorption of chloride ions and formaldehyde by CGs in cement concrete applications. At the same time, we will expand the area of reuse of CGs and improve the recycling rate of CGs.

## 2. Materials and Methods

### 2.1. Raw Materials

**Figure 1:**
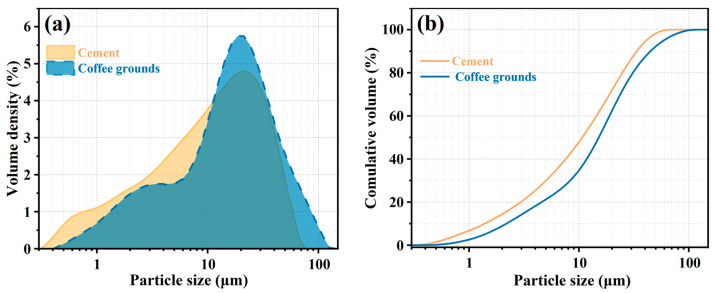
Particle size distribution curve and cumulative distribution curve of cement and CGs. (**a**) volume density; and (**b**) cumulative volume.

The P·O 42.5 ordinary Portland cement utilized in this study was sourced from Yunnan Huaxin Cement Factory. The chemical composition of this cement is detailed in Table 1. CGs were obtained from a Starbucks cafe in Yunnan, subjected to a washing process to eliminate surface impurities, and subsequently dried in an oven at 60 °C for 24 h to prevent mold formation. The dried CGs were placed into a ball mill and ground until they passed through a 200-mesh sieve. Subsequently, they were sealed for later use. The chemical composition of CGs is provided in Table 1. Due to their significant organic content, the loss on ignition (LOI) value is notably high. The chemical composition of CGB is presented in Table 1, with the key components being P_2_O_5_, Fe_2_O_3_, and SO_3_. The particle size distribution of cement and ground CGs is illustrated in Figure 1. The median particle sizes for cement and CGs are 12.2 μm and 17.2 μm, respectively.

**Figure 2:**
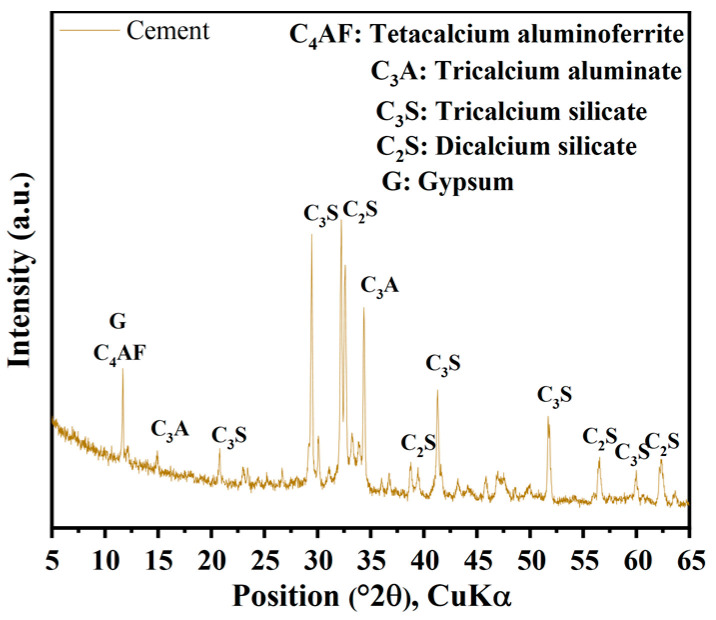
XRD patterns of cement.

**Figure 3:**
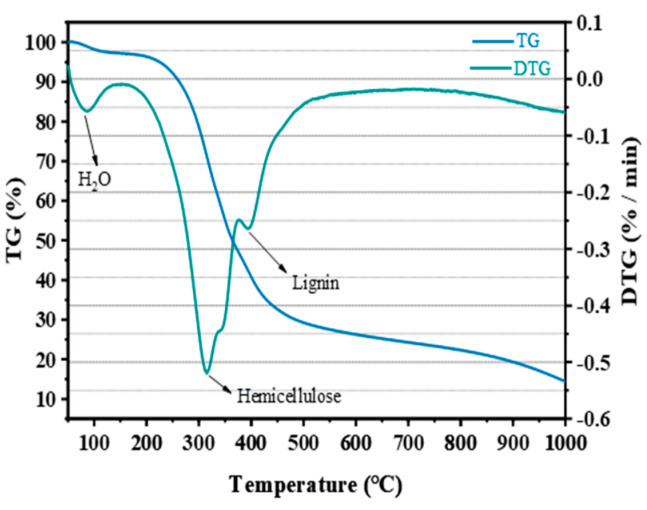
TG−DTG curve of CGs.

Figure 2 displays the X-ray diffraction (XRD) patterns of cement. It is evident from the figure that the mineral composition of cement primarily includes C_3_S, C_2_S, C_3_A, C_4_AF, and gypsum. The TG-DTG analysis of CGs in Figure 3 indicates that CGs are predominantly composed of organic compounds, encompassing hemicellulose, cellulose, lignin, and so forth.

Furthermore, a portion of the sieved CGs was immersed in a 50% phosphoric acid solution for modification over a 24 h period, maintaining a CG/phosphoric acid mass ratio of 1:3. Once the impregnation process was completed, the material was placed in a constant-temperature drying oven at 95 °C for 24 h. After drying, the two layers were enveloped with tin foil and placed in a muffle furnace. The temperature was gradually increased to 450 °C at a rate of 8 °C/min, which was maintained for 2 h, and then the layers were allowed to cool to room temperature. Subsequently, they were washed repeatedly with distilled water until the pH was neutral and then dried at 95 °C for 24 h to obtain CGB.

### 2.2. Mix Ratio and Sample Preparation

To investigate the impact of CGs and CGB on the adsorption performance of cementitious materials, we employed equal proportions of 0%, 2%, 4%, 6%, and 8% CGs and CGB to substitute cement in the preparation of test specimens. Based on the results of the standard-consistency water consumption test [11], a water/cement ratio (water/(cement + CGs/CGB)) of 0.29 was utilized. The mix ratio design is detailed in Table 2.

### 2.3. Preparation of the Samples

The dimensions of the clean slurry specimen were 25 × 25 × 25 mm. The specimen preparation process was conducted according to the guidelines outlined in GB/T17671-2021 [12]. Due to the low water/cement ratio and poor fluidity, the slurry was poured into the test mold twice to prevent the formation of additional pores. After pouring each layer, the mold was placed on a vibrating table to ensure thorough compaction through vibration. The specimen mold was cured in a standard curing room with a temperature of 20 ± 2 °C and a relative humidity exceeding 95% for a period of 1 day. Subsequently, the mold was removed.

After the specimen was demolded, it underwent further curing in the standard curing room for 28 and 90 days.

### 2.4. Testing Methods

#### 2.4.1. Compressive Strength

After curing the specimens for 28 and 90 days, a compressive strength test was conducted. Each group consisted of six parallel specimens, and the reported strength value represents the average for the six specimens.

#### 2.4.2. Chloride Ion Adsorption

Under identical conditions, another set of clean slurry specimens for chloride ion adsorption was prepared. In this case, water was replaced with a 0.55 M sodium chloride solution, introducing chloride ions into the specimen. Following curing under identical conditions for 28 and 90 days, the specimens were fractured, and internal fragments were chosen and ground into a powder until they could pass through a 0.6 mm sieve. Subsequently, the powder samples were placed in a 105 °C oven for 2 h to allow them to dry. Afterward, they were taken out and transferred to a vacuum drying dish to allow them to cool to room temperature for future use. The total chloride ion content (C_t_) and the quantity of free chloride ions (C_f_) in the sample were tested in accordance with SL T352-2020 [13], and the adsorption rate of chloride ions by the sample was calculated according to Formula (1).

(1) $$Chloride\;ion\;adsorption\;rate=\frac{C_{t}-C_{f}}{C_{t}}$$

#### 2.4.3. Formaldehyde Adsorption

Upon curing the clean slurry specimen for 90 days, a formaldehyde adsorption test was conducted according to the procedures outlined in QBT2761-2006 [14]. The experiment was conducted in a test chamber with a volume of 1 m^3^. The test chamber was partitioned into blank test chamber A and sample test chamber B. Two test specimens were placed in test chamber B. We prepared two 500 mL reagent bottles and filled them with 200 mL of formaldehyde pollutant with 0.2% concentration. Two glass rods, each enveloped in 5 layers of gauze, were vertically placed into the two reagent bottles, designating them as release sources A_1_ and A_2_. Release sources A_1_ and A_2_ were placed in blank test chamber A and sample test chamber B, respectively, and the doors were closed promptly. The fans in cabins A and B were activated, stirring was applied for 1 min, and then the fans were turned off. After a lapse of 24 h, air sampling tests were conducted in cabins *A* and *B*, followed by analysis. The recorded formaldehyde concentrations in the air were denoted as *C_A_* and *C_B_*, respectively. The formaldehyde removal rate (γ) was calculated according to Formula (2).

(2) $$\gamma =\frac{C_{A}-C_{B}}{C_{A}}$$

#### 2.4.4. Product Test Analysis

Following the fracturing of the specimen, internal fragments were chosen and immersed in absolute ethanol for 3 days to halt hydration. Subsequently, they were removed and placed in a vacuum-drying dish to dry them for future use. A portion of the fragments was ground and sifted through a cement sieve for composition testing and analysis, while another portion was preserved for microscopic morphology observation. XRD, TG−DTG, BET, FTIR, SEM, and XPS technologies were employed for testing and analyzing the product components.

An automatic X-ray diffractometer (Ultima IV X-ray radiation diffractometer, Tokyo, Japan) was employed to capture X-ray diffraction patterns and determine the crystal composition of the sample. The instrument utilized a copper target radiation with continuous scanning, operating at a wavelength of 0.128796 nm, voltage of 40 kV, current of 40 mA, and scanning speed of 5°/min.

A synchronous thermal TG−DTG system was utilized to investigate the influence of CG and CGB content on cement hydration products. Employing a Japanese HITACHI STA200 thermogravimetric analyzer (Hitachi, Tokyo, Japan), the weight loss of the sample during heat treatment was monitored. The analysis was conducted under inert nitrogen protection conditions, with a heating rate of 15 °C/min, a temperature range from 50 °C to 1000 °C, and a N_2_ flow rate of 50 mL/min.

Utilizing the American Micromeritics APSP 2460 model 4-station (Micromeritics Instrument Corporation, Norcross, GE, USA) fully automatic specific surface area analyzer, under 77 k liquid nitrogen conditions, each sample underwent a nitrogen adsorption and desorption test to obtain an isothermal adsorption and desorption curve. The curve was analyzed using the BET method to determine the total specific surface area of the material.

FTIR infrared spectra of the samples were collected using the German Bruker Tensor 27 (Bruker, Karlsruhe, Germany) instrument, with a resolution of 4 cm^−1^ and 32 scans. The testing range covered 500–4000 cm^−1^.

To analyze the hydration products and micromorphological characteristics of the samples, a scanning electron microscope (ZEISS GeminiSEM 300, ZEISS, Jena, Germany) was employed. The microstructural characteristics of the samples were examined under an accelerating voltage of 15 kV.

For testing, an X-ray photoelectron spectrometer (XPS), Model Thermo Scientific K-Alpha (Thermo Fisher Scientific, Wilmington, DE, USA), was utilized. This instrument identified the elemental characteristics of the material through Al Kα rays (hv = 1486.6 eV).

## 3. Results and Discussion

### 3.1. Comparison of XRD, FTIR, and BET between CGs and CGB

**Figure 4:**
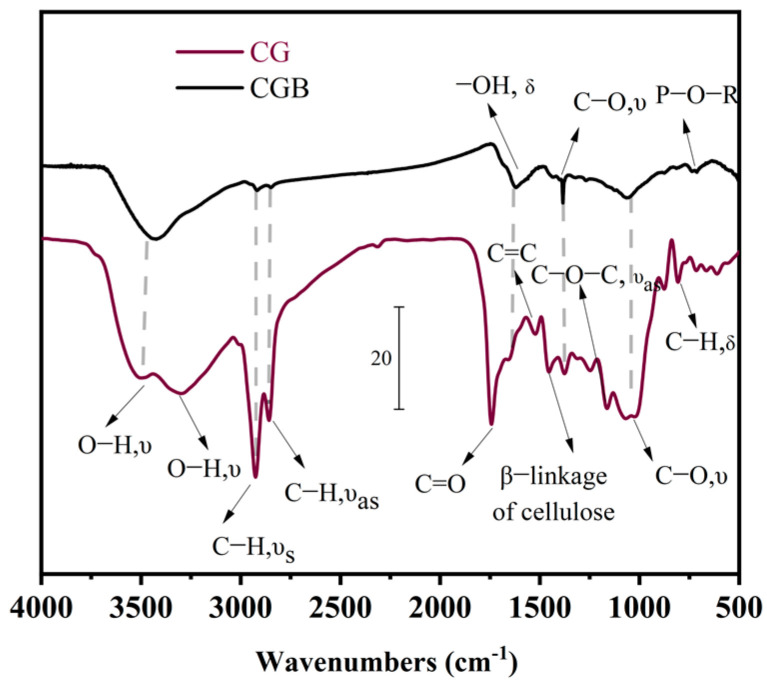
FTIR curves of CGs and CGB.

The XRD diffraction peak pattern of CGs reveals distinct dispersion peaks between 10° and 30°, primarily attributed to the presence of hemicellulose, lignin, and amorphous cellulose [15]. Fourier transform infrared spectroscopy (FTIR) analysis (Figure 4) confirmed the presence of cellulose (873cm^−1^, 1375 cm^−1^, and 1460 cm^−1^) [16] and lignin (1530 cm^−1^) [17,18], in addition to the presence of substances such as caffeine (2680 cm^−1^ and 2925 cm^−1^) [16].

**Figure 5:**
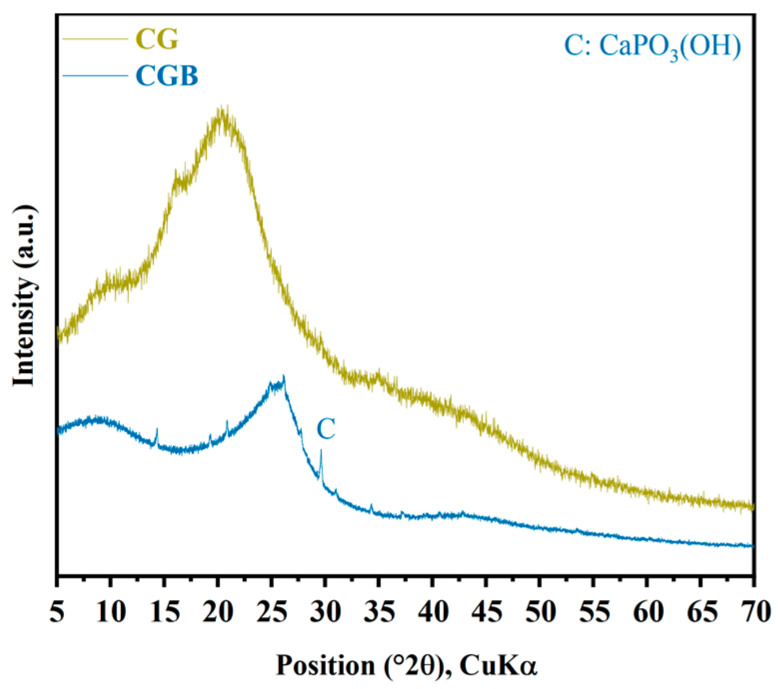
XRD patterns of CGs and CGB.

**Figure 6:**
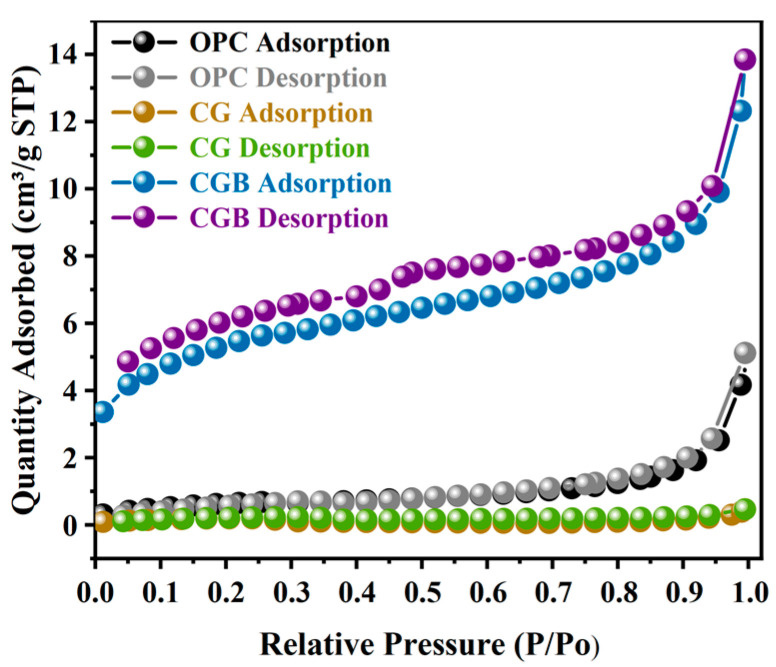
Nitrogen adsorption–desorption isotherms of cement, CGs, and CGB.

The XRD pattern of CGB is depicted in Figure 5. Similar to CGs, CGB also displays characteristic broad diffraction bands within the range of 2θ = 10−30°. This is primarily attributed to the presence of amorphous carbon compounds, such as lignin and hemicellulose [19]. In comparison with CGs, the amorphous hump intensity of CGB diminishes and shifts to a higher 2θ angle, particularly one concentrated around 26°. This alignment with the typical peak characteristics of pure carbon suggests that the residual carbon after the pyrolysis of CGs exists in the form of pure carbon in CGB [20]. Furthermore, a distinct CaPO_3_(OH) crystal peak was identified in the CGB. At the same time, it can also be seen in Table 1 that CGB contains a large amount of P_2_O_5_. In the FTIR analysis (Figure 4), it became evident that in comparison to CGs, CGB exhibits pronounced absorption bands solely at 3420 cm^−1^, 1050 cm^−1^, and 1375 cm^−1^, with the disappearance of the remaining absorption bands. This is primarily attributed to the fact that a substantial portion of the organic compounds in CGs underwent decomposition under the influence of high temperatures during the pyrolysis process. The absorption peak at 1050 cm^−1^ can be ascribed to the P−O−R bond formation resulting from the reaction between H_3_PO_4_ and CGs. This is similar to the results observed by Ma et al. [19]. According to the Brunauer–Emmet–Teller measurements of the specific surface area for CGs and CGB (Figure 6), it is evident that the specific surface area of CGB is significantly greater than that of CGs. This indicates that the phosphoric acid high-temperature pyrolysis of CGs results in a distinct pore structure and augments the specific surface area of CGB. Molina-Sabio et al. also found a similar phenomenon [4]. As evident from the Supplementary Material (Tables S1−S3), CGB exhibits a larger pore volume and smaller pore diameter than CGs.

### 3.2. Compressive Strength

**Figure 7:**
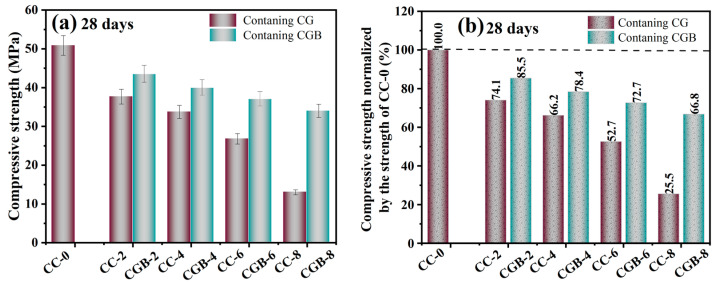

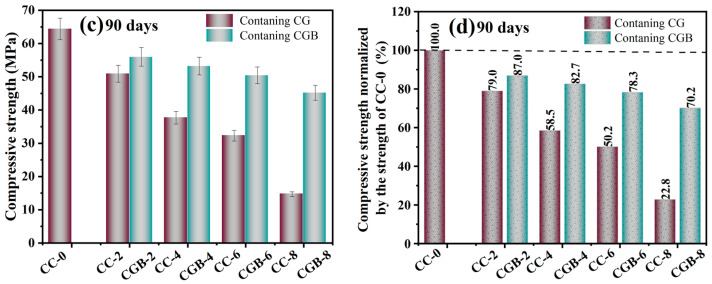
Compressive strength: (**a**) compressive strength of mortars at 28 days; (**b**) compressive strength of mortars normalized to the strength of CC-0 at 28 days; (**c**) compressive strength of mortars at 90 days; (**d**) compressive strength of mortars normalized to the strength of CC-0 at 90 days.

**Figure 8:**
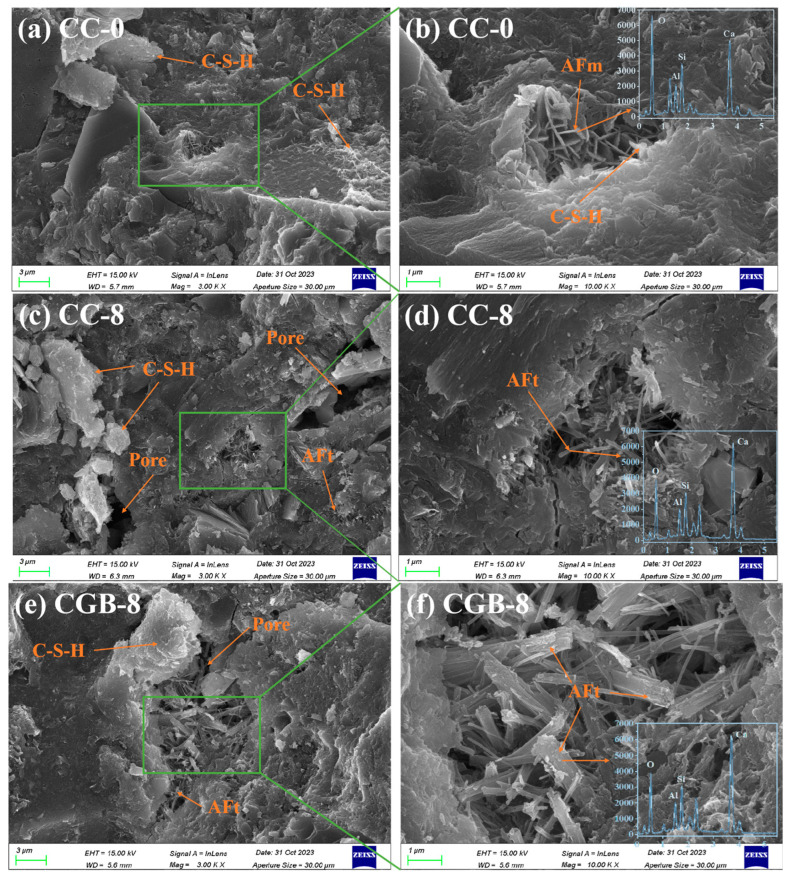
(**a**) SEM images of the CC-0 sample after 28 days; (**c**) SEM images of the CC-8 sample after 28 days; (**e**) SEM images of the CGB-8 sample after 28 days; (**a**,**c**,**e**) represent SEM images magnified 3000 times, respectively; (**b**,**d,f**) represent SEM images magnified 10,000 times, respectively.

**Figure 9:**
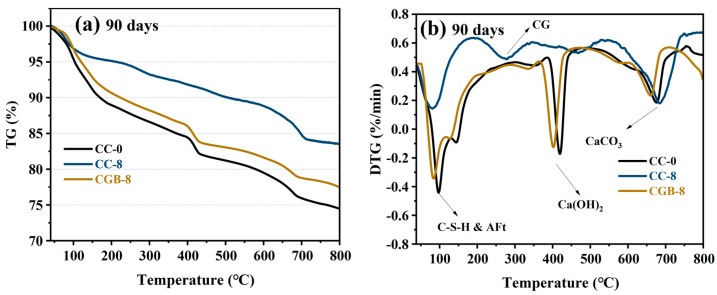
TG−DTG curves of cement pastes containing different CG and CGB contents: (**a**) TG curve of the sample after curing for 90 days; (**b**) DTG curve of the sample after curing for 90 days.

**Figure 10:**
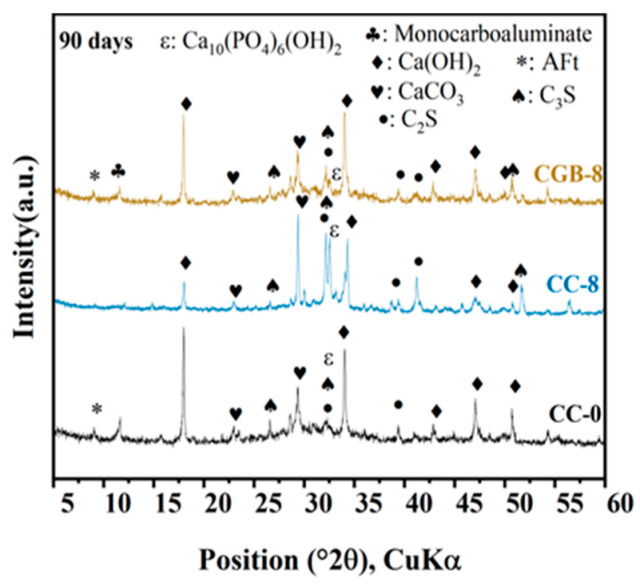
XRD patterns of cement pastes containing different CG and CGB contents.

Figure 7 illustrates the impact of CGs and CGB content on the compressive strength of cement slurry specimens at 28 and 90 days. It is evident from the figure that the inclusion of both CGs and CGB has a detrimental impact on the strength of cement. Moreover, as the quantity increases, the strength consistently decreases. This trend aligns with findings reported by Fonseca et al. [21] and Roychand et al. [20]. This phenomenon can be attributed to the following factors: (1) The strength of the composite system is primarily derived from cement hydration products. The addition of CGs and CGB introduces a dilution effect, diminishing the formation of hydration products, and hinders the development of cement strength. (2) With increasing amounts of CGs and CGB, the actual water/cement ratio (water/cement) in the system rises. The experiments indicate a significant deterioration in the working performance of the mixture as well. All of these factors influence the compactness of cementitious materials to a certain extent. Figure 8 shows the SEM images and EDS spectra of the CC-0, CC-8, and CGB-8 samples after curing for 28 days. It can be observed from the microscopic image that the main hydration products in the sample are needle-like ettringite crystals, flocculent or amorphous C−S−H, and flake-like monosulfide salts. In addition, we observed that there were significantly more pores in the cc-8 sample, which were not filled by hydration products. This may also be one of the reasons for the reduction in compressive strength. (3) The organic compounds in CGs did not effectively participate in product formation but significantly impeded the hydration reaction of cement, especially the hydration of C_3_S and C_2_S. The TG−DTG curve (refer to Figure 9) indicates that the main hydration products such as C-S-H gel and Ca(OH)_2_ were not produced sufficiently in the CC-8 sample, and the organic compounds from the original CGs still persist in the CC-8 sample. Generally speaking, the greater the amount of chemically bound water (Wb) and Ca(OH)_2_ in the hardened cement, the higher the degree of hydration and the more hydration products [22]. As shown in Table 3, the results indicate that the content of chemically bound water (Wb) and Ca(OH)_2_ decreases in the following order: CC-0 > CGB-8 > CC-8. This is consistent with its strength development results. The XRD pattern (Figure 10) also confirms that C_3_S and C_2_S still clearly exist in the CC-8 sample. (4) The XRD pattern (Figure 10) reveals that the P_2_O_5_ in CGB reacts with the Ca^2+^ in the cement to form hydroxyapatite Ca_10_(PO_4_)_6_(OH)_2_. This reaction results in a deficiency of Ca^2+^ and hinders the nucleation and growth of colloids in the C-S-H, impacting its condensation [23] and resulting in a reduction in its compressive strength.

On the other hand, the detrimental impact of CGB on the strength of the cement slurry specimens was not as pronounced as that of CGs. This was primarily attributed to the decomposition of organic compounds through the pyrolysis of CGs, mitigating the adverse impact of organic compounds on cement hydration. The TG−DTG curve (refer to Figure 9) indicates that the main hydration products such as C−S−H gel and Ca(OH)_2_ in the CGB-8 sample have effectively formed, and there is virtually no presence of organic compounds. The XRD pattern (Figure 10) further validates that there are essentially no unhydrated particles such as C_3_S and C_2_S in the CGB-8 sample. Additionally, the pore volume of CGs increased after phosphoric acid pyrolysis treatment, refer to the Supplementary Material (Tables S1−S3). The actual water/cement ratio of the gelling system mentioned in the previous paragraph can be reduced to a certain extent through water absorption [20,24]. Furthermore, the experiment revealed that the working performance of the cement slurry mixture containing CGB was notably superior to that of the mixture containing CGs. All of these factors contributed to an improvement in the compactness of the CGB cement slurry specimen to a certain extent (refer to Figure 8) and mitigated the strength loss caused by the reduction in cement content.

### 3.3. Chloride Ion Adsorption

**Figure 11:**
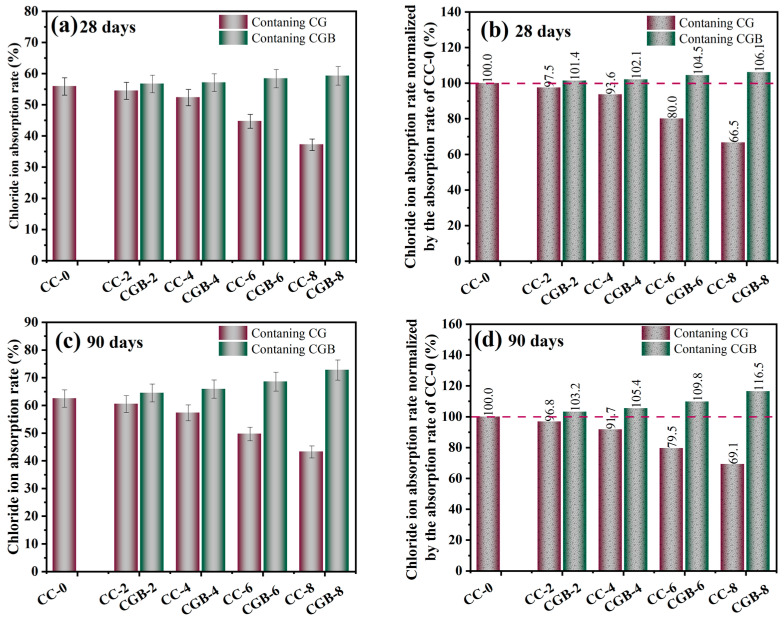
Chloride ion adsorption rate of pastes: (**a**) chloride ion adsorption rate at 28 days; (**b**) 28-day chloride ion adsorption rate of mortars normalized by the adsorption rate of CC-0; (**c**) chloride ion adsorption rate at 90 days; (**d**) 90-day chloride ion adsorption rate of mortars normalized by the adsorption rate of CC-0.

**Figure 12:**
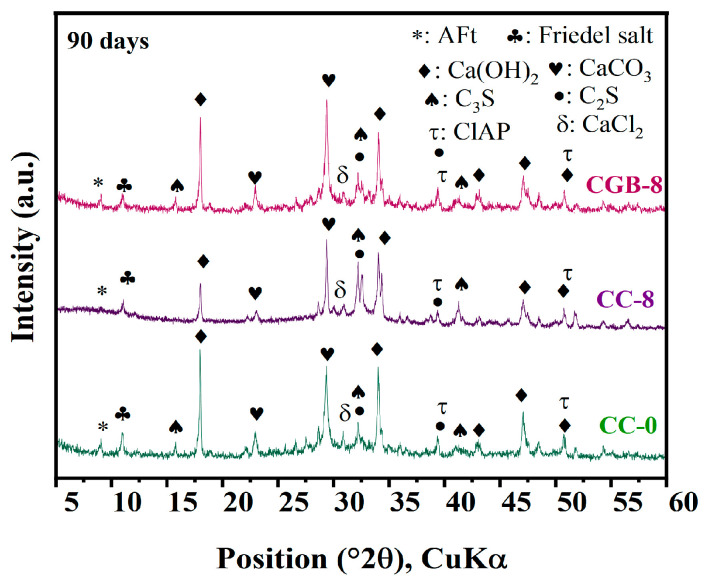
XRD pattern under the action of chlorine salt.

The chloride ion adsorption rates of the clean slurry specimens at 28 and 90 days are illustrated in Figure 11. It is evident from the figure that with the increase in the amount of CGs, the chloride ion binding rate of the clean slurry specimen exhibits a decreasing trend at both 28 and 90 days. It is well known that the adsorption of chloride ions by cement primarily occurs through the reaction of C_3_A and chloride ions, leading to the formation of FS, as well as the physical combination of C−S−H gel. The XRD pattern (Figure 12) reveals the presence of FS in the CG sample. However, as analyzed in Section 3.2, the inclusion of CGs significantly impedes the formation of C−S−H gel [25]. Therefore, it is speculated that the inclusion of CGs primarily affects the physical adsorption of chloride ions by C−S−H gel, thereby reducing the chloride ion adsorption rate of cement.

Conversely, the inclusion of CGB improved the chloride ion adsorption rate of the cement slurry specimens, with the adsorption rate increasing as the amount of CGB increased. On the one hand, after CGB was treated with phosphoric acid and high temperatures, the hindrance of cement hydration weakened, allowing for the smooth generation of hydration products to ensure efficient adsorption. On the other hand, as evident from the BET test results in the Supplementary Material (Tables S1−S3), CGB after phosphoric acid activation treatment was conducive to the formation of micropores during the pyrolysis process. Simultaneously, the specific surface area and pore volume increased, leading to a corresponding adsorption performance enhancement [4]. Furthermore, after CGB combined with cement, thus forming Ca_10_(PO_4_)_6_(OH)_2_, chloride ions and OH^−^ ions underwent replacement, leading to a chemical reaction that forms chlorapatite [26]. However, the corresponding adsorption mechanism still requires further research for clarification.

### 3.4. Formaldehyde Adsorption

**Figure 13:**
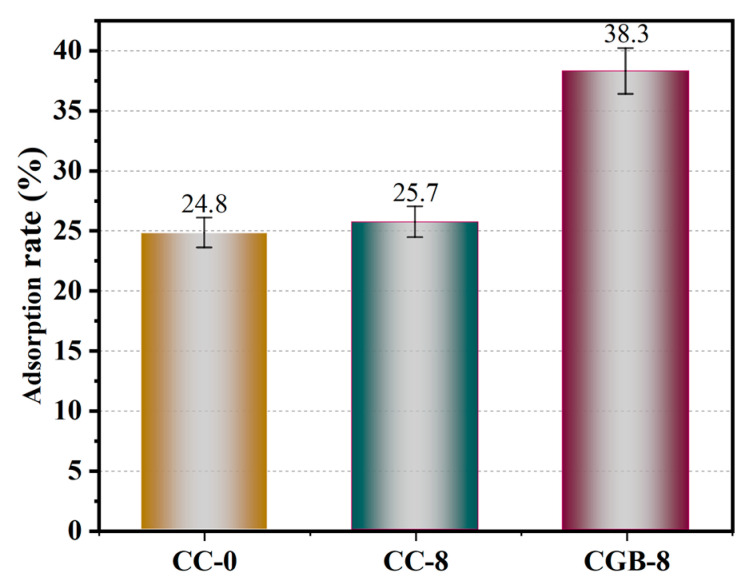
Formaldehyde adsorption rate.

Figure 13 presents the formaldehyde adsorption results for specimens CC-0, CC-8, and CGB-8 at 90 days of age. It is evident from the figure that the inclusion of both CGs and CGB enhances the formaldehyde adsorption capacity of cement slurry specimens. The improvement effect of CGs is relatively weaker, but the enhancement effect of CGB is more pronounced. The adsorption effect of cement on formaldehyde is attributed to the electronegative nature of formaldehyde molecules, making them easily adsorbable on the surface of positively charged C−S−H gel. On the other hand, cement can absorb and solidify formaldehyde through its own pores. Subsequently, it combines with hydroxyl radicals in the air, undergoes oxidation into formic acid, and finally decomposes into H_2_O and CO_2_ [27]. The inclusion of CGs severely restricts the generation of cement hydration products, yet the formaldehyde adsorption capacity does not decrease accordingly. It is evident that CGs have a robust adsorption capacity for formaldehyde, compensating for the adverse impact on cement. On the one hand, the cellulose, hemicellulose, and lignin structures in CGs can effectively bind with formaldehyde molecules through hydroxyl and carboxyl functional groups [28]. On the other hand, the porous structure of CGs also facilitates the adsorption of formaldehyde within it [29].

**Figure 14:**
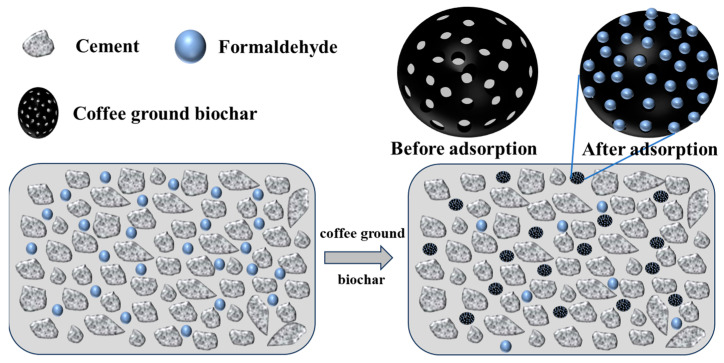
Formaldehyde adsorption mechanism diagram.

**Figure 15:**
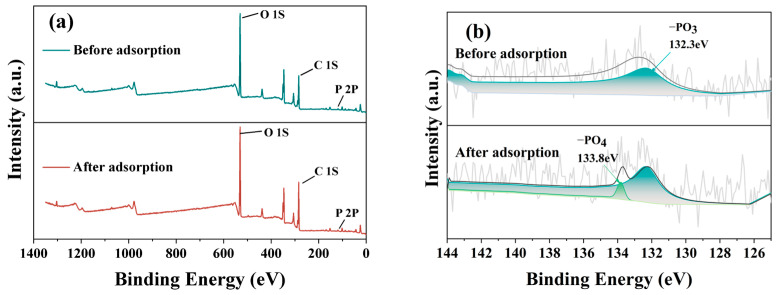
XPS patterns of CGB−8 sample before and after formaldehyde adsorption at 28 days: (**a**) XPS patterns before and after formaldehyde adsorption; (**b**) XPS patterns of P 2p before and after adsorption.

After CGs undergo phosphoric acid high-temperature pyrolysis, a significant portion of the organic compounds is decomposed, and the remaining hydroxyl groups play a crucial role in the adsorption of formaldehyde. This is because formaldehyde is a hydrophilic molecule, and the hydroxyl group, as a hydrophilic functional group, better facilitates the absorption of formaldehyde molecules on hydrophilic surfaces [30]. The formaldehyde adsorption mechanism is shown in Figure 14, which more vividly explains the adsorption process of formaldehyde molecules on hydrophilic surfaces. Moreover, after the treatment with phosphoric acid and high temperatures, the phosphorus-containing groups separate the organic compounds in CGs, causing the structure to expand and endowing it with a high-quality pore structure [31]. As indicated by the BET results in the Supplementary Material (Tables S1–S3), the specific surface area and pore volume of CGB significantly increased, and the pores became more refined, thereby enhancing the adsorption capacity for formaldehyde. It is noteworthy that the XPS results (Figure 15) reveal changes and shifts in the P 2p peak of the sample before and after the adsorption of formaldehyde molecules. The broad peak ranging from BE = 536.08 to 526.08 eV is attributed to the (C=O=C) or (C=H) bond [19,32], while the broad peak at BE = 291.08-281.08 eV corresponds to the (C=C) or (C−H) bond [33,34]. The peak at BE = 132.3 eV represents the C−PO_3_(−PO_3_) bond [19,35,36], and BE = 133.8 eV represents the C−O−PO_3_(−PO_4_) bond. Notably, comparing samples before and after formaldehyde adsorption reveals changes and shifts in the p 2p peak. Additionally, a new peak labeled C−O−PO_3_(−PO_4_) appears at 133.8 eV in samples adsorbing formaldehyde [19,33,35]. This may be attributed to the adsorption of formaldehyde molecules by phosphorus-containing groups in CGB, linking the oxygen in formaldehyde to the C−PO_3_(−PO_3_) bond. Consequently, after CGs are activated into CGB through pyrolysis of phosphoric acid, its adsorption performance is significantly improved, consistent with the adsorption rate results. Considering that the inclusion of CGB has little effect on the hydration of cement, the adsorption of formaldehyde by both components in the cement−CGB composite system has been effectively realized. Under the dual effect, the CGB-8 specimen exhibits the highest adsorption capacity for formaldehyde, showcasing optimal adsorption performance. In addition, the CC-0 sample did not undergo phosphoric acid pyrolysis treatment. The CC-0 sample lacks the adsorption effect of P groups on formaldehyde. Therefore, we did not perform XPS analysis on the CC-0 sample.

## 4. Conclusions

According to the experimental framework of this study, the following conclusions can be drawn:

1. Since organic compounds seriously hinder the hydration reaction of cement, the incorporation of CGs limits the strength development of cement. A significant amount of organic compounds in CGB was eliminated through pyrolysis, and its negative impact on cement strength was not as pronounced as in the case of CGs. However, due to the dilution effect and the competition between hydroxyapatite and calcium ions, the strength development of cement was still limited to a certain extent.
2. Since hydration products were not effectively generated, the inclusion of CGs limited the cement’s adsorption of chloride ions. However, the incorporation of CGB had a minor effect on cement hydration. Moreover, during the pyrolysis process, a large number of fine pores formed, increasing the specific surface area. Considering the replacement effect of hydroxyapatite, the chloride ion adsorption effect was improved.
3. CGs are porous and rich in hydroxyl and carboxyl functional groups. When mixed into cement, they enhance the adsorption of formaldehyde by the cement. The improvement effect of CGB is more pronounced, and this can mainly be attributed to its more optimized pore structure and richness in hydroxyl and phosphorus-containing groups, promoting the adsorption of formaldehyde.

## Figures and Tables

**Table 1:** Chemical composition of cement, CGs, and CGB (wt.%).

Raw Material	CaO	SiO_2_	Al_2_O_3_	MgO	Fe_2_O_3_	SO_3_	MnO	K_2_O	BaO	P_2_O_5_	LOI
Cement	63.14	20.34	5.25	2.96	2.8	2.6	1.56	-	-	-	1.35
CGs	0.438	0.027	-	0.159	0.13	0.24	-	0.174	0.079	0.073	98.68
CGB	0.370	0.424	0.135	0.10	28.865	3.26	0.722	0.024	-	65.55	0.55

**Table 2:** Mix proportion design.

No.	Cement (wt.%)	CG (wt.%)	CGB (wt.%)
CC-0	100	0	-
CC-2	98	2	-
CC-4	96	4	-
CC-6	94	6	-
CC-8	92	8	-
CGB-2	98	-	2
CGB-4	96	-	4
CGB-6	94	-	6
CGB-8	92	-	8

**Table 3:** Chemically bound water (Wb) and portlandite (Ca(OH)_2_) contents.

Specimen	Time (Days)	Wb (wt.% Cement Mass)	Ca(OH)_2_ (wt.% Cement Mass)
CC-0	90	24.3	16.42
CC-8	90	12.2	8.06
CGB-8	90	21.2	14.62

## Data Availability

The data presented in this study are available from the corresponding author upon reasonable request.

## Supplementary Materials

The following supporting information can be downloaded at: https://www.mdpi.com/article/10.3390/ma17040907/s1, Table S1: Comparison with surface area detection results (m^2^/g), Table S2: Pore volume analysis results (cm^3^/g), Table S3: Pore size analysis results (nm).
