# Supplementary material for: Effect of Coffee Grounds/Coffee Ground Biochar on Cement Hydration and Adsorption Properties
Source: Materials (Basel). 2024 Feb 15;17(4):907. doi: 10.3390/ma17040907 (PMC10890603; doi:10.3390/ma17040907)
Supplement: Supplementary file 1 [file materials-17-00907-s001.zip › materials-2845939-supplementary.pdf]

materials

MDPI

sSupporting information

Effect of Coffee Grounds / Coffee Ground biochar on Cement Hydration and Adsorption Properties

Yang Chen[1,2,3], Rong-xin Guo[1,2,3], Fei-yue Ma[1,2,3], Hao-xue Zhou[1,2,3], Miao Zhang[1,2,3], Qian-min Ma[1,2,3,*]

[1] Faculty of Civil Engineering and Mechanics, Kunming University of Science and Technology, Kunming 650500, China
[2] Yunnan Key Laboratory of Disaster Reduction in Civil Engineering, Kunming 650500, China
[3] International Joint Laboratory for Green Construction and Intelligent Maintenance of Yunnan Province, Kunming 650500, China
* Correspondence: maqianmin666@163.com (Qian-min Ma)

Keywords: Chloride ion adsorption ability; Formaldehyde adsorption ability; Compressive strength; Coffee ground biochar; Cement

Table S1. Comparison with surface area detection results (m²/g).

Sample	BET Surface Area	Langmuir Surface Area	T-Plot external Surface Area
OPC	2.5217	13.8643	1.4537
CG	0.8044	0.6778	1.1120
CGB	19.4195	47.4442	17.7514

Table S2. Pore volume analysis results (cm³/g).

Sample	Total pore volume	T-Plot micropore volume	BJH Adsorption cumulative volume	BJH Desorption cumulative volume
OPC	0.0079	0.0003	0.0078	0.0078
CG	0.0007	0.0002	0.0006	0.0006
CGB	0.0214	0.0007	0.0173	0.0162

Table S3. Pore size analysis results (nm).

Sample	Average pore diameter	BJH Adsorption average pore	BJH Desorption average pore
OPC	12.5487	15.3155	14.7409
CG	3.5963	9.1697	5.2937
CGB	4.4116	6.8381	6.2581
